# Impaired fasting glucose, single-nucleotide polymorphisms, and risk for colorectal cancer in Koreans

**DOI:** 10.4178/epih/e2016002

**Published:** 2016-01-06

**Authors:** Keum Ji Jung, Miyong To Kim, Sun Ha Jee

**Affiliations:** 1 Department of Public Health, Graduate School, Yonsei University, Seoul, Korea; 2 School of Nursing, The University of Texas at Austin, Austin, TX, USA; 3 Institute for Health Promotion and Department of Epidemiology and Health Promotion, Graduate School of Public Health, Yonsei University, Seoul, Korea

**Keywords:** Impaired fasting glucose, Single-nucleotide polymorphisms, Colorectal cancer

## Abstract

**OBJECTIVES:**

Numerous studies have demonstrated that fasting serum glucose (FSG) levels and certain single-nucleotide polymorphisms (SNPs) are related to an increased risk of colorectal cancer (CRC); however, their combined effects are still unclear.

**METHODS:**

Of a total of 144,527 men and women free of cancer at baseline, 317 developed CRC during 5.3 years of follow-up. A case-cohort study (n=1,691) was used, consisting of participants with a DNA sample available. Three well-known SNPs (rs3802842, rs6983267, rs10795668) were genotyped. Hazard ratios (HR) and 95% confidence intervals (CI) of CRC, colon and rectal cancer were calculated, with the Cox proportional hazard models.

**RESULTS:**

The crude incidence rates per 100,000 person-years were 41.1 overall, 48.4 for men, and 29.3 for women. Among participants with dysglycemia, SNPs rs3802842 and rs6983267 were both associated with an increased risk of CRC (HR, 3.2; 95% CI, 1.9 to 5.5 and HR, 1.8; 95% CI, 1.1 to 3.1, respectively) and rectal cancer (HR, 3.4; 95% CI, 1.8 to 6.6 and HR, 3.3; 95% CI, 1.6 to 7.1, respectively). The interaction effect of dysglycemia and SNPs was positive, that is, resulted in an elevated risk of CRC, but was not statistically significant.

**CONCLUSIONS:**

This study demonstrates that both high FSG and certain SNPs are major risk factors for CRC and rectal cancer but that they did not interact synergistically. The difference in effect size of the SNPs according to CRC subtype (i.e., colon or rectal cancer) and presence of dysglycemia merits further research.

## INTRODUCTION

Colorectal cancer (CRC) is one of the most common cancers worldwide. In Korea, the standardized incidence of CRC has increased from 21.2/100,000 people in 1999 to 57.6/ 100,000 people in 2012 [1]. A steady increase in the incidence of CRC is expected in the future, partly as a result of factors such as the increase in Western dietary patterns, the increase in diabetes [2], and interaction between these factors and genetic factors [3]. In particular, an association with several common single-nucleotide polymorphisms (SNPs) has been reported, with high replicability. The SNPs associated with CRC have been found to account for 4.3% of the variation in multiple adenoma risk, with three SNPs (rs3802842, rs6983267, rs10795668) explaining 3.0% of the variation [3].

Although colonoscopy plus biopsy is the gold standard for CRC screening and diagnosis, more than half of all patients do not want to undergo this procedure because of the invasive nature and intestinal discomfort of colonoscopy [4]. Compared to endoscopy, tests of serum biomarkers are more convenient and less invasive and can be better accepted as part of a routine physical examination [5], but the diagnostic value of most serum CRC markers still remains poor for most patients [6].

Although many epidemiologic studies of CRC have investigated diabetes and genetics as risk factors, there has been a limited exploration of their combined effects, reflecting limitations posed by the study population size and the exposure data available in the studies carried out to date. Detecting synergism among common risk factors would have substantial public health and clinical relevance because it would identify some individuals who are at extremely high risk. It is well known that early discovery of high-risk groups is beneficial in reducing CRC incidence and mortality by enabling management of risk factors [7].

We have conducted a prospective cohort study of the causes of cancer in Koreans. Blood samples and information concerning diabetes were available for all participants in this large cohort. Follow-up was accomplished through record linkage at the national level and was complete except for emigrants. Here, we describe the risk for CRC in relation to fasting serum glucose (FSG) and SNPs over 5.3 years of follow-up, during which there were 234 diagnoses of CRC among our cohort of 117,258.

## MATERIALS AND METHODS

### Study subjects

**Figure 1.**
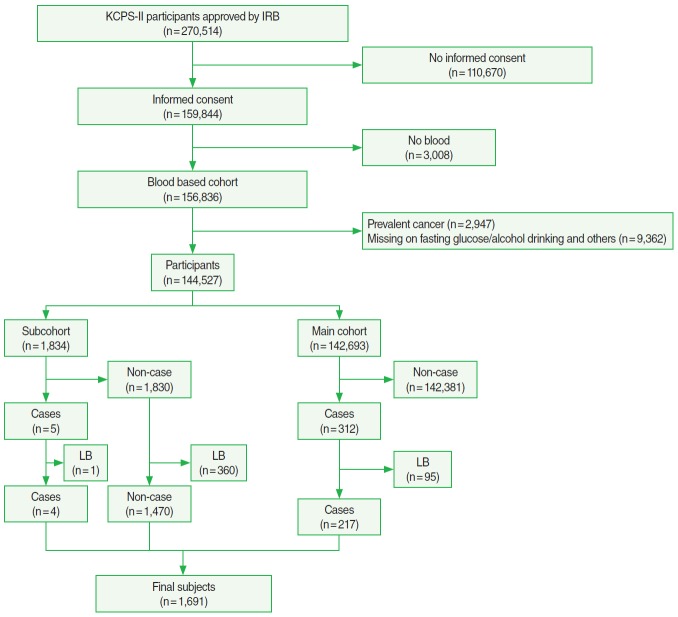
Flow chart describing sample size. KCPS-II. Korean Cancer Prevention Study-II; IRB, institutional review board; LB, limited amount of blood.

Participants for this study were drawn from a pool of 270,514 individuals (153,372 men and 117,142 women) who had voluntarily undergone health examinations in 11 centers located in Seoul and Kyunggi provinces in Korea from 1994 to 2010, which were used as the baseline. Of these individuals, 159,844 provided informed consent and were followed prospectively, forming the cohort of participants in the Korean Cancer Prevention Study-II (KCPS-II). A full description of KCPS-II has been previously published [2]. Of the 159,844 participants, 156,836 donated a blood specimen for genetic studies. Of the 156,836 participants, 2,947 who reported having prevalent cancer, including prevalent CRC, were excluded. In addition, 9,362 participants who had missing values for body mass index (BMI), fasting blood glucose, smoking status, alcohol drinking, and/or exercise were excluded. As of December 2012, 317 of these 144,527 participants had been diagnosed with CRC. For our case-cohort study, we selected a subcohort for genetic testing, which constituted an about 1.3% random sample (n=1,834) of all 144,527 participants (Figure 1). The institutional review board of Yonsei University approved the study.

### Questionnaire and anthropometric measurements

Each participant was interviewed by using a structured questionnaire to collect the following details: smoking history (never smoked, ex-smoker, or current smoker), alcohol drinking (non-drinker vs. consumer of any amount of alcohol on a regular basis), and regular exercise (yes or no). Participant’s height and weight were measured while the participants were wearing light clothing. BMI was calculated by dividing the weight (kg) by the square height (m2). Systolic and diastolic blood pressures were measured after a rest period of at least 15 minutes.

### Blood collection and biochemical analyses

For clinical chemistry assays, serum (separated from peripheral venous blood) was obtained from each participant after a minimum fasting period of 12 hours and stored at -70°C until the time of analysis. Levels of FSG, total cholesterol, triglyceride, low-density lipoprotein cholesterol, and high-density lipoprotein cholesterol were measured by using a Cobas Integra 800 (Roche Diagnostics, Rotkreuz, Switzerland) and a Hitachi-7600 analyzer (Hitachi, Tokyo, Japan).

### Single-nucleotide polymorphisms genotyping

As genetic factors for the risk of CRC, three well-known SNPs (rs3802842 in the COLCA1 gene, rs6983267 in the CASC8 gene, and rs10795668 in the LOC105376400 gene) were selected from previous studies [3]. DNA samples were isolated from the peripheral blood of participants and genotyped at DNA Link Inc. (Seoul, Korea). The genotyping was performed using a SNP typing assay (Fluidigm, San Francisco, CA, USA) according to the manufacturer’s instructions. The genomic DNA flanking the SNPs of interest was amplified by polymerase chain reaction (PCR) with the specific target amplification (STA) primer set and Qiagen 2X Mutiplex PCR Master Mix (Qiagen, Hilden, Germany) in a 5-μL reaction volume containing 60 ng of genomic DNA. The PCR reactions were carried out as follows: 15 minutes at 95ºC hot start, and 14 cycles of 1) 95ºC for 15 seconds, 2) 60ºC for 4 minutes. After amplification, the STA products were diluted 1:100 in DNA suspension buffer. A sample (2.5 μL) of each diluted STA product was added to a Sample Pre-Mix containing 3 μL of 2X Fast Probe Master Mix, 0.3 μL of the SNPtype 20X Sample Loading Reagent, 0.1 μL of the SNPtype Reagent, and 0.036 μL of the ROX Reference Dye (Cat. no. 12223-012; Invitrogen Ltd., Paisley, UK). After the Assay Pre-Mix and the Sample Pre-Mix were loaded onto the 48.48 Dynamic Array, the SNP typing assay reaction was carried out. Analysis was carried out using Fluidigm SNP Genotyping Analysis software (version 4.0.1; Fluidigm Corp., South San Francisco, CA, USA). Internal quality control measures were employed to ensure the accuracy of the data. A total of 1,691 individuals were genotyped via this platform.

### Diagnosis of colorectal cancer

Data regarding the incidence of CRC were obtained from the records of the National Cancer Registry. All incidence of CRC was confirmed by histological cell type. According to the International Classification of Diseases, 10th revision (ICD-10), CRC was coded as C18-C20 [9]. Deaths from CRC were ascertained from the cause of death listed on death certificates.

### Statistical analysis

Person-years were calculated from the baseline enrollment to December 2012 or the date of CRC diagnosis, death, or loss to follow-up. Crude incidence rates (per 100,000 person-years) were calculated from the person-years and number of cases of CRC. Hazard ratios (HRs) were calculated by using the Cox proportional hazard model, after adjusting for age, sex, BMI, smoking status, alcohol consumption, and regular exercise. These analyses were used after the FSG levels were divided into four categories on the basis of cut-offs at 100 mg/dL, 110 mg/dL, and 126 mg/dL. A chi-square goodness-of-fit test was used to assess whether the SNPs were in Hardy-Weinberg equilibrium and to identify differences in genotype frequencies between CRC cases and controls. All statistical tests were two-sided, and statistical significance was determined as p<0.05. SAS version 9.2 (SAS Institute Inc., Cary, NC, USA) was used for all analyses.

## RESULTS

The basic characteristics of the participants (144,527 in the whole cohort and 1,834 in the subcohort) are summarized in Table 1 and Figure 1. A total of 317 participants were newly diagnosed with CRC among the 144,527 participants over 771,052 total person-years (mean follow-up 5.3 years) through to December 2012. The crude incidence rates per 100,000 person-years were 41.1 overall, 48.4 for men, and 29.3 for women. The population had a low BMI on average, with 39.5% of the men and 15.1% of the women at 25 kg/m^2^ or above and 3.5% of the men and 1.6% of the women above 30 kg/m^2^. Both smoking and alcohol use were substantially more common in men than in women.

Table 2 shows the age-adjusted rates and relative risks (RRs) of CRC in relation to FSG and the three common SNPs. Higher FSG was associated with an increased risk of CRC, particularly among subjects with impaired fasting glucose (IFG; 100 ≤FSG ≤125 mg/dL). The findings were similar when we examined the relationship of FSG to CRC subgroup among the subjects with DNA data. Considering only those participants with DNA (SNP) data, we examined the potential relationships between genotypes and CRC risk. The RR for CRC for those with genotype AC or CC in SNP rs3802842 was 1.7 and 1.3 times higher, respectively, than for those with genotype AA. The RRs for colon cancer and rectal cancer were similar, with the same genotypic pattern for SNP rs3802842. Also, those with genotype TT in SNP rs6983267 had an increased risk of rectal cancer (HR, 2.3; 95% CI, 1.3 to 3.9), but not colon cancer. The RR for colon cancer with genotype GG in SNP rs10795668 was significant (HR, 1.9; 95% CI, 0.9 to 3.9).

Table 3 shows the age-adjusted and gender-adjusted combined effect of SNPs and dysglycemia on the risk of CRC. Among participants with dysglycemia, both SNPs rs3802842 and rs6983267 were associated with an increased risk of CRC (HR, 3.2; 95% CI, 1.9 to 5.5 and HR, 1.8; 95% CI, 1.1 to 3.1, respectively) and rectal cancer (HR, 3.4; 95% CI, 1.8 to 6.6 and HR, 3.3;

95% CI, 1.6 to 7.1, respectively). The interaction effect of dysglycemia and SNPs was positive, resulted in an elevated risk of CRC, but was not statistically significant.

## DISCUSSION

In this large prospective cohort study of the Korean population, we found that high FSG and SNPs rs3802842 and rs6983267 were independent risk factors for CRC, colon, and rectal cancer. No interaction between these risk factors was observed.

### Hyperglycemia

In particular, we found that hyperglycemia, including IFG, was associated with CRC incidence, indicating that pre-diabetic status might be related to colorectal carcinogenesis. This finding was consistent with those of several previous studies [2,8], although the specific classification and study designs differed between the studies. In the Framingham Heart Study offspring cohort, the incidence of CRC was found to be higher in subjects with IFG. The early exposure to high glucose levels was strongly associated with CRC, and the HRs proportionally increased with the length of the exposure to IFG [9]. There are many mechanistic hypotheses to account for this association between hyperglycemia and CRC, including hyperinsulinemia occurring through insulin-like growth factor signaling [10-12], chronic inflammation [12,13], delayed bowel transit time [14], changes in bile acid [15], and imbalanced microbiota in the bowel [16].

### Genetic factors

When considering early exposure to risk factors for a disease, genetic factors are obvious candidates because exposure begins before birth and continues for an entire lifetime. In the present study, the incidence of rectal cancer was associated with both SNPs rs3802842 and rs6983267 [3]. The underlying mechanism of how these SNPs are associated with the incidence of colon cancer is as yet undetermined. The association between SNP rs3802842 and CRC has been replicated in at least seven previous studies, with consistent positive associations [17]. Also, SNP rs6983267 has been replicated in at least 14 previous studies in Western populations [18]. Both SNPs have been previously reported in Asian populations but only SNP rs6983267 was found to be associated with rectal cancer in the present study. In contrast, SNP rs10795668 did not show any association with CRC, colon, or rectal cancer incidence in our study. Previous studies were carried out as genome-wide association studies designating CRC as the outcome [19]. These studies found an association between CRC and CRC-related SNPs. Thus, only CRC in general has been examined, and colon and rectal cancer had not yet been studied separately as targets. However, in our study the SNPs that were associated with rectal cancer showed less correlation with colon cancer. This finding suggests that future association studies should look separately at colon and rectal cancers as well as CRC and progression. It follows that prediction models for colon and rectal cancer should also be developed.

Our study has several potential limitations that arise primarily from the use of data collected as part of routine medical examinations. The statistical power of the current study may have been too low, since genotyping was performed only in a limited sample. In addition, our study cohort was not necessarily representative of all Koreans because it included only employed persons and their families. However, follow-up should be almost complete because of our use of record linkage with unique personal identifiers to national databases; thus, any loss to follow-up is unlikely to have introduced bias.

Our study has analyzed the possibility of a mutual interaction of diabetes and genetic factors as a potential predictor of CRC, and is of importance to the public health and health promotion sectors. Research efforts should continue to analyze the interactions between genetic factors, environmental risk factors, and prognostic factors that are associated with CRC.

## Figures and Tables

**Table 1.** General characteristics of study participants: the Korean Cancer Prevention Study-II

		Whole cohort (n = 144,527)	Subcohort (n = 1,834)
Age (yr)		41.2±10.3	40.7±9.9
BMI (kg/m^2^)^1^		23.6±3.2	23.5±3.1
FSG (mg/dL)		91.4±19.1	90.3±17.6
Total cholesterol (mg/dL)		189.2±33.8	189.2±40.8
Systolic blood pressure (mmHg)		117.9±14.4	117.5±14.5
Sex (female, %)		38.3	36.6
Conditions (%)			
Dysglycemia (FSG >110 mg/dL)		8.6	6.8
Any alcohol use		74.1	75.5
Exercise		58.5	60.6
Family history^2^		2.4	2.5
Smoking status (%)			
Ex-smokers		17.6	16.5
Current smokers		28.9	28.7
Genetic polymorphism			
rs3802842	AA		35.4
	AC		48.9
	CC		15.7
rs6983267	GG		19.0
	GT		48.5
	TT		32.6
rs10795668	AA		11.8
	AG		48.2
	GG		39.9

BMI, body mass index; FSG, fasting serum glucose.

1 BMI=weight in kg divided by height in m^2^.

2 Family history of colorectal cancer

**Table 2.** Risk factors for colorectal cancer incidence as analyzed by Cox proportional hazard models: the Korean Cancer Prevention Study-II

		Whole cohort (n=144,527)			Subjects with DNA data (n=1,691), case-cohort design					
					Colorectal cancer		Colon cancer		Rectal cancer	
		Case	Rate^1^	RR (95% CI)	Case	RR (95% CI)	Case	RR (95% CI)	Case	RR (95% CI)
Age (yr)		317	41.1	1.1 (1.1, 1.1)	221	1.1 (1.1, 1.1)	114	1.1 (1.1, 1.2)	107	1.1 (1.1, 1.1)
Gender	Men	230	48.4	1.0 (reference)	163	1.0 (reference)	82	1.0 (reference)	81	1.0 (reference)
	Women	87	29.4	0.7 (0.5, 0.8)	58	0.6 (0.4, 0.8)	32	0.6 (0.4, 1.0)	26	0.5 (0.3, 0.8)
FSG (mg/dL)	<100	205	32.8	1.0 (reference)	142	1.0 (reference)	73	1.0 (reference)	69	1.0 (reference)
	100-109	53	67.9	1.4 (1.0, 1.9)	37	1.1 (0.8, 1.6)	20	1.2 (1.0, 2.8)	17	1.1 (0.7, 1.9)
	110-125	22	77.8	1.3 (0.9, 2.0)	15	1.8 (1.2, 2.6)	9	1.7 (1.0, 2.8)	6	1.9 (1.1,3.2)
	≥126	37	93.8	1.2 (0.9, 1.8)	27		12		15	
Genetic polymorphism										
rs3802842	AA				60	1.0 (reference)	33	1.0 (reference)	27	1.0 (reference)
	AC				124	1.7 (1.2, 2.3)	61	1.5 (1.0, 2.3)	63	2.0 (1.3, 3.1)
	CC				37	1.3 (0.8, 2.0)	20	1.3 (0.7, 2.4)	17	1.4 (0.8, 2.6)
rs6983267	GG				53	1.0 (reference)	25	1.0 (reference)	28	1.0 (reference)
	GT				102	1.1 (0.8, 1.5)	47	0.8 (0.5, 1.2)	55	1.7 (1.0, 2.7)
	TT				66	1.4 (1.0, 2.0)	42	0.9 (0.5, 1.5)	24	2.3 (1.3, 3.9)
rs10795668	AA				22	1.0 (reference)	9	1.0 (reference)	13	1.0 (reference)
	AG				99	1.0 (0.6, 1.6)	56	1.4 (0.7, 2.9)	43	0.7 (0.4, 1.3)
	GG				100	1.4 (0.8, 2.2)	49	1.9 (0.9, 3.9)	51	1.1 (0.6, 1.9)

RR, relative risk; CI, confidence interval; FSG, fasting serum glucose.

1 Age-adjusted rate per 100,000 person years.

**Table 3.** Age- and gender-adjusted combined effect of SNP rs3802842 and dysglycemia on the risk of colorectal cancer among subjects with SNP data (n=1,369)

		Colorectal cancer		Colon cancer		Rectal cancer	
		Case	RR (95% CI)	Case	RR (95% CI)	Case	RR (95% CI)
Dysglycemia	rs3802842						
No	AA	51	1.0 (reference)	29	1.0 (reference)	22	1.0 (reference)
No	AC or CC	128	1.5 (1.1, 2.1)	65	1.3 (0.9, 2.1)	67	1.9 (1.2, 3.0)
Yes	AA	9	1.6 (0.7, 3.4)	4	1.3 (0.4, 3.8)	5	2.1 (0.7, 5.9)
Yes	AC or CC	33	3.2 (1.9, 5.5)	17	2.5 (1.3, 4.6)	16	3.4 (1.8, 6.6)
p-value for interaction			<0.001		0.007		< 0.001
Dysglycemia	rs6983267						
No	GG	47	1.0 (reference)	32	1.0 (reference)	16	1.0 (reference)
No	GT or TT	132	1.4 (1.0, 2.0)	62	1.0 (0.6, 1.5)	73	2.3 (1.3, 4.0)
Yes	GG	19	3.4 (2.0, 5.8)	11	2.8 (1.4, 5.7)	8	4.2 (1.8, 9.9)
Yes	GT or TT	23	1.8 (1.1,3.1)	10	1.1 (0.5, 2.3)	13	3.3 (1.6, 7.1)
p-value for interaction			0.001		0.29		< 0.001
Dysglycemia	rs10795668						
No	AA	20	1.0 (reference)	9	1.0 (reference)	12	1.0 (reference)
No	AG or GG	159	1.2 (0.7, 1.9)	85	1.5 (0.7, 3.0)	77	0.9 (0.5, 1.6)
Yes	AA	2	3.0 (0.7, 12.8)	0	0.0	2	4.6 (1.0, 20.4)
Yes	AG or GG	40	2.1 (1.2, 3.6)	21	2.5 (1.1,5.6)	19	1.6 (0.8, 3.4)
p-value for interaction			0.001		0.014		0.05

SNP, single-nucleotide polymorphisms; RR, relative risk; CI, confidence interval.
